# Semi-Precise Analytical Method for Investigating the Liftoff Variation on the Hall Sensor in Metal Defect Sensing

**DOI:** 10.3390/s21165539

**Published:** 2021-08-17

**Authors:** Ali Azad, Jong-Jae Lee, Namgyu Kim

**Affiliations:** 1 Department of Civil and Environmental Engineering, Sejong University, Seoul 05006, Korea; g2016sfd008@sju.ac.kr (A.A.); jongjae@sejong.ac.kr (J.-J.L.); 2 Department of Structural Engineering Research, Korea Institute of Civil Engineering and Building Technology, Goyang 10223, Korea

**Keywords:** damage detection, magnetic flux leakage, Hall sensor, liftoff, numerical analysis, experimental analysis

## Abstract

Hall-effect sensors are used to detect metal surface defects both experimentally and numerically. The gap between the specimen and the sensor, called the liftoff, is assumed to remain constant, while a slight misplacement of a sample may lead to incorrect measurements by the Hall-effect sensor. This paper proposes a numerical simulation method to mitigate the liftoff issue. Owing to the complexity of conducting precise finite-element analysis, rather than obtaining the induced current in the Hall sensor, only the magnetic flux leakage is obtained. Thus, to achieve a better approximation, a numerical method capable of obtaining the induced current density in the circumferential direction in terms of the inspection direction is also proposed. Signals of the conventional and proposed approximate numerical methods affected by the sensor liftoff variation were obtained and compared. For small liftoffs, both conventional and proposed numerical methods could identify notch defects, while as the liftoff increased, no defect could be identified using the conventional numerical method. Furthermore, experiments were performed using a variety of liftoff configurations. Based on the results, considering the threshold of the conventional numerical method, defects were detected for greater liftoffs, but misdetection did not occur.

## 1. Introduction

All structures have a service life during which time they should be able to withstand certain loads and weather conditions. A damaged structure may not be able to fulfill the anticipated load capacity, possibly causing partial or total failure of the structure. To prevent structural failure, structures should undergo regular inspections. Initially, non-destructive testing (NDT) started with basic visual inspection [1] and gradually improved over time. Currently, many more advanced defect-detection techniques exist. Conventional health-monitoring methods that take advantage of propagating wave phenomena can be classified as follows: mechanical wave-based techniques (e.g., ultrasonic guided waves [2,3] and acoustic emission [4,5]) and electromagnetic wave-based techniques (e.g., magnetic flux leakage (MFL) [6,7] and eddy current testing [8,9]). Among the different types of NDT health-monitoring techniques, the MFL inspection method is one of the most popular inspection methods for identifying both surface and internal defects [10]. This method is capable of identifying corrosion [11], cross-sectional damage [12], axial cracks [13], circumferential cracks [14], and internal defects [15].

Kim and Park [16] proposed a damage detection and quantification method using signal processing in the case of an MFL Hall sensor. In their approach, damage indexes were obtained for a variety of artificial notches with multiple depths, widths, and lengths by applying an enveloping process to the signals obtained from Hall sensors. Park et al. [17] developed a cable-climbing robot using an MFL sensor probe that contained an array of Hall sensors arranged circumferentially, and the performance of the apparatus was experimentally investigated. Xiucheng et al. [18] designed and produced a tailor-made tunnel magnetoresistive (TMR) device with sensors mounted in a circular array. To address the missed detection issue of axial cracks in the pipelines, Wu et al. [13] proposed using an alternating magnetic field excitation. Their apparatus was a combination of alternating current induction coils and permanent magnets along with an array of Hall sensors. Wang et al. [19] investigated the effects of high-speed inspections on Hall sensor signals. They observed that, by increasing the specimen’s speed, the amplitude of the MFL signal increased also.

Recently, owing to the rapid advancement of finite-element commercial software programs capable of simulating a variety of problems with reliable results, many researchers have begun to conduct numerical analyses prior to experiments. This is because an approximation of the experiment’s outcome should reduce the required trial-and-error process, ultimately leading to an optimized solution in terms of both the time and total cost of the experiment. Currently, the main aim of conventional numerical simulation is to obtain changes in the MFL signal when a specimen is defective. Yu et al. [20] performed a parametric optimization through conventional finite-element analysis (FEA) and using a genetic algorithm to obtain the optimum dimensions of the sensor head. Gao et al. [14] compared the MFL testing method with the eddy current pulsed thermography method. In a conventional numerical investigation, the orientation and depth of cracks were investigated, and the flux leakage was visualized and plotted. Liu et al. [21] proposed a circumferential excitation method to identify axial cracks in pipelines. Based on two-dimensional (2D) conventional numerical simulations, axial defects were detected correctly. To overcome the overheating issue caused by direct current (DC) in the solenoid, Liu et al. [15] developed a sensor probe that works with a biased pulse current and identified both surface and subsurface defects. However, in their numerical simulation, the input current of the solenoid was DC, and only the MFL signal corresponding to the DC was obtained. Liu et al. [22] evaluated the effects of a weak magnetic field on a ferromagnetic sample. They performed a conventional numerical simulation and validated the simulation results by performing experimental analyses. Wu et al. [23] investigated the effects of the discontinuity orientation and the scanning direction of the sensor. Ma et al. [24] improved the MFL signal-to-noise ratio (SNR) by implementing a magnetic concentrating device in the vicinity of the Hall sensor. Further, signal enhancement was approved by performing both conventional numerical and experimental analyses. The sensitivity enhancement of the MFL signal was investigated by Wu et al. [25] using a ferromagnetic support. The efficiency of ferromagnetic support was confirmed based on both numerical simulation and experiment results. Suresh et al. [26] designed an MFL probe that can fit inside a small-diameter steam generator tube. Suitable dimensions of the magnetizer unit were numerically obtained and used in the experiment. However, enhancing the signal intensity by implementing a highly permeable ferromagnetic material may alter the output signal of the sensor owing to the magnetic hysteresis effect and residual magnetism in a ferromagnetic enhancer unit. Wu et al. [27] developed an MFL probe containing a TMR sensor and Helmholtz-like coils. Furthermore, a 2D conventional numerical simulation was performed to obtain the optimum dimensions of the sensor head.

Wu et al. [7] introduced a liftoff tolerant inspection method. To detect surface flaws, they placed an array of coil sensors circumferentially and proposed to amplify the signal intensity by adding a ferrite core. The influence of the ferrite core on the signal was investigated both numerically and experimentally. Dutta et al. [28] conducted a three-dimensional (3D) conventional numerical simulation to assess the effects of the defect’s shape and liftoff on the MFL signal. To do so, they developed a liftoff compensation technique to predict the defect size. However, the considered liftoff values were limited to a maximum of 10 mm. Feng et al. [29] investigated the effects of the sensor liftoff variation due to unwanted vibrations during inspection. Moreover, a mapping algorithm was proposed to enhance the SNR of the MFL signal, and its influence on the signal was assessed both numerically and experimentally. Nonetheless, only the effects of vibration during the inspection on signal quality were assessed and improved. Sun et al. [30] designed an open magnetizing unit capable of reducing the magnetic interaction force while generating a more uniform magnetic field. Furthermore, the magnetizing unit of the apparatus was optimized using a conventional numerical method. However, the sensor liftoff was assumed to be constant, and, hence, its influence on the MFL was not studied.

Prior to conducting an experimental test, a numerical study through FEA can be performed to provide a reasonable approximation of the test outcome. However, precise numerical analyses require a sophisticated multiphysics analysis of charged particles and electromagnetic physics. Owing to the high level of complexity of this numerical analysis, a simplified numerical analysis capable of obtaining the MFL was employed in previous studies. This numerical analysis was conventionally performed to evaluate the MFL in the vicinity of a defect. Due to the MFL in the region of interest being acquired by defining a reference line in the surrounding air, and it is complicated to model the Hall sensor as well as the induced Hall voltage based on the Hall-effect phenomenon, the Hall sensor is not modeled in the conventional numerical analysis. Consequently, in a numerical simulation in which the Hall sensor is disregarded, the interaction between the magnetic field and electric field in the Hall sensor is disregarded also.

By modeling the Hall sensor’s geometry through a numerical analysis, the current density generated in the Hall sensor by the interaction between the Hall sensor’s geometry and MFL can be evaluated. Thus, to overcome the limitation of conventional numerical techniques in evaluating the induced Hall voltage, this paper also proposes a numerical approximation method capable of estimating induced current density in the circumferential direction with respect to the inspection direction. In particular, rather than obtaining the MFL value, the objective of the numerical simulation was to acquire the circumferential induced current density. To achieve this goal, a conductive plate (i.e., a simplified model of the Hall sensor) in a 2D axisymmetric space through time-dependent analysis was defined and simulated. Subsequently, the effects of liftoff variation in both the conventional and proposed approximate numerical methods were established and compared with each other. Finally, the effects of the liftoff variation were investigated experimentally.

The remainder of this paper is organized as follows. The mechanics of the Hall sensor are briefly explained in Section 2. In Section 3, numerical simulations, including conventional and proposed numerical simulations along with liftoff variation effects, are explained in detail. The experimental evaluation is presented in Section 4, and the conclusions of this research are presented in Section 5.

## 2. Theoretical Background

### 2.1. Theory of the Hall-Effect Sensor

**Figure 1:**
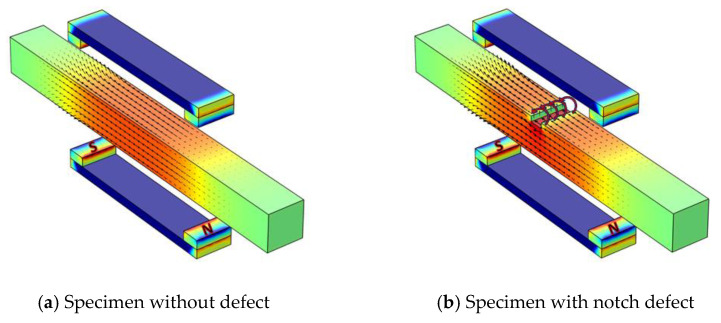
Schematic of the MFL phenomenon.

**Figure 2:**
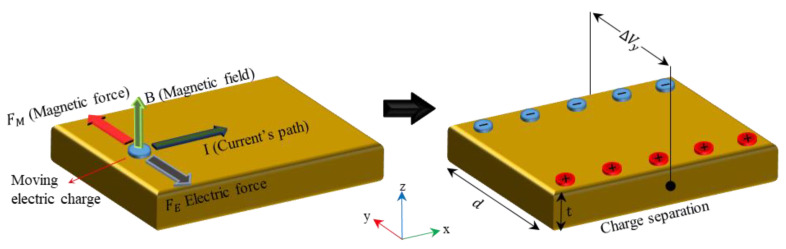
Charge separation in a conductive plate due to the Hall-effect phenomenon.

When a defective sample is exposed to a magnetic field strong enough to induce magnetization near saturation, an MFL appears around the defect. The leakage of magnetic flux from a sample into the surrounding boundaries is called the MFL. It should be noted that the necessary condition for the MFL-based method is that the magnetizing source must provide a magnetic field strong enough to magnetize the sample up to the saturation point [10,30]. Figure 1 presents the schematic MFL when the specimen is magnetized up to its saturation point. The leaked magnetic flux can be detected by a Hall sensor mounted in the correct direction. In this study, a Hall sensor was mounted in the direction in which its sensitive axis was aligned in the direction of the radial component of the magnetic flux. Hall sensors work based on the Hall-effect phenomenon, where an electrically conductive plate with a known electric current running through the plate in a direction parallel to the *x*-axis is placed in a magnetic field perpendicular to the current direction (i.e., parallel to the *z*-axis). Owing to the electromagnetic force, which is governed by the Lorentz force law, charge separation occurs on opposite sides of the conductive plate (i.e., the *y*-axis direction). This charge separation leads to a voltage difference perpendicular to the current direction, and this voltage difference is called Hall voltage. This phenomenon is illustrated in Figure 2.

### 2.2. Proposed Method: Approximate Time-Dependent Numerical Simulation

**Figure 3:**
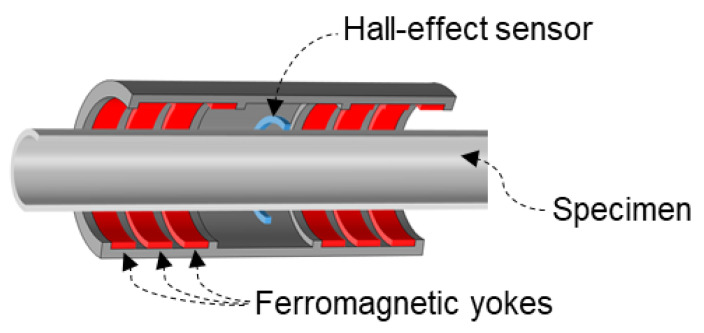
Schematic cut-section of the simulation model.

A numerical simulation of the Hall sensor is generally performed as a guideline prior to the experimental test. In conventional numerical methods, a Hall sensor is disregarded in the numerical model, whereas only a reference line above the region of interest is defined to acquire the MFL signal. It should be noted that, by disregarding the geometry of the Hall sensor in the numerical simulation, the interaction between the magnetic field and electric field is inevitably ignored. As a result, only the MFL signal corresponding to defects can be obtained; more importantly, the conventional simulation is not capable of acquiring any current induction. In this study, a semi-realistic FEA was performed, while the geometry of the Hall sensor was not disregarded in the numerical model. Nonetheless, it is computationally expensive to simulate charged particles under the influence of magnetic force considering the actual sensor scale using FEA. Hence, rather than simulating the exact Hall-effect phenomenon, an approximate numerical approach based on the eddy current phenomenon was proposed in this study. To satisfy this aim, the circumferential induced current density in the Hall sensor should be numerically evaluated. Acquiring the circumferential induced current density requires three steps, compared to the technique used in the conventional numerical model. First, rather than defining a reference line in the air domain, the Hall sensor geometry is defined and modeled as a conductive plate. Second, rather than performing a static simulation, a time-dependent simulation must be performed. The reason for choosing a time-dependent simulation over a static simulation is that current induction cannot be acquired in a static simulation. In a time-dependent simulation, not only is it possible to consider and simulate the effect of the specimen’s motion, but, owing to the time dependency of the relationship between the magnetic field and electric field (governing Maxwell’s equation), current induction caused by MFL can be measured accordingly. Third, rather than performing a 2D simulation, a 3D or 2D axisymmetric simulation should be performed to acquire circumferential current induction. In this study, a 2D axisymmetric simulation was performed. Figure 3 shows a schematic view of a pipe sample along with the sensing unit used in both the numerical analysis and experimental tests.

### 2.3. Governing Equations

#### 2.3.1. Hall-Effect Phenomenon

When conducting numerical analyses based on the electromagnetic theory for a conductive plate and a ferromagnetic material representative of the Hall sensor and ferromagnetic specimen, respectively, Maxwell’s equations and the Lorentz force law (Equation (1)) are the governing equations. Initially, charge separation does not occur, and the electric field is therefore equal to zero. Thus, the magnetic force acting on the electric charges can be obtained using Equation (2).

(1) $$F=q\left(E+v_{D}\times B\right)$$

(2) $$E=0,\cdots ,\;F_{M}={\mathrm{qv}}_{D}\times B$$

where F, q, E, $v_{D}$, B, and $F_{M}$ are the electromagnetic force, electric charge, electric field, drift velocity, magnetic flux density, and magnetic force, respectively. After charge separation occurs, the electric charges are subjected to an electric force, which is governed by Coulomb’s law (Equation (3)). Eventually, charge separation reaches equilibrium, which emphasizes that both electric and magnetic forces have the same magnitude. Thus, in the equilibrium condition, the voltage difference generated in a conductive plate can be obtained using Equation (5).

(3) $$F_{E}={\mathrm{qE}}_{H}$$

(4) $$F_{M}=F_{E}\to E_{H}=v_{D}B$$

(5) $$V_{H}=E_{H}d=v_{D}\mathrm{Bd}$$

where $F_{E}$, $E_{H}$, $V_{H}$, and d are the electric force, Hall’s electric field, Hall’s voltage difference, and the width of the conductive plate, respectively. According to the charge conversion principle, the drift velocity can be obtained using Equation (6). Finally, by substituting Equation (6) into Equation (5), a simplified form of the Hall voltage difference can be obtained using Equation (7).

(6) $$I_{\mathrm{ave}}=\frac{\Delta Q}{\Delta t},\Delta Q=q\left(\mathrm{nA}\Delta x\right),\Delta x=v_{D}\Delta t\to v_{D}=\frac{I_{\mathrm{ave}}}{\mathrm{nqA}}$$

(7) $$V_{H}=\frac{I_{\mathrm{ave}}B}{\mathrm{nqt}}$$

where $I_{\mathrm{ave}}$, Q, n, A, x, and t are the average current, charge transferred through the cross- section over time, charge carrier density, cross-sectional area of the conductive plate, length of the conductive plate, and thickness of the conductive plate, respectively.

#### 2.3.2. Proposed Analytical Method

Similar to the Hall-effect phenomenon, Maxwell’s equations are the governing equations in the case of current induction based on the eddy current phenomenon. In addition, Ohm’s law (Equation (8)) was used to derive the induced current density. Thus, for a moving magnetized specimen with an MFL value of $B_{\mathrm{MFL}}$ and velocity v, the induced current density in a fixed conductive plate is obtained using Equation (9).

(8) J=σE

(9) $$J_{i}=\sigma \left(E+v\times B_{\mathrm{MFL}}\right)+J_{e},$$

where J, σ,${\;J}_{i}$, v, and $J_{e}$ are the current density, electrical conductivity, induced current density, velocity of the specimen, and external current density, respectively.

## 3. Numerical Simulation

### 3.1. Description of the Simulation Setup

**Figure 4:**
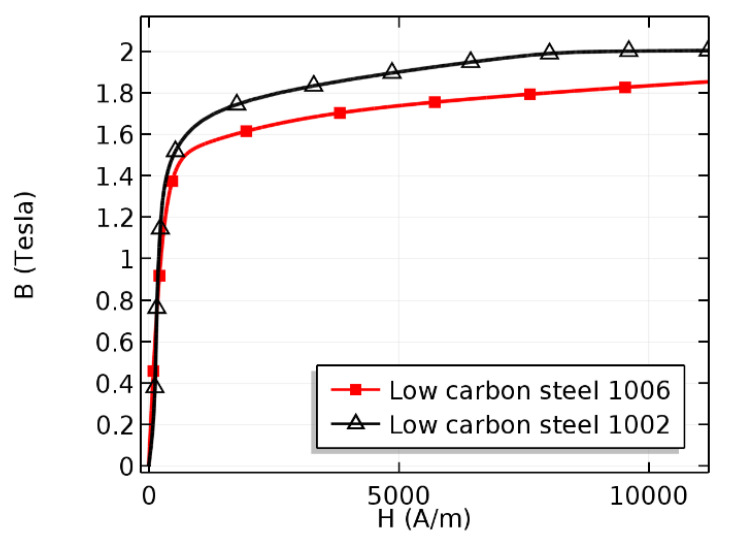
BH curve of low-carbon steel 1002 and 1006.

To investigate the effects of varying the liftoff, conventional and proposed precise simulation models were prepared and simulated using COMSOL Multiphysics, as shown in Figure 3. In particular, to consider the electromagnetic physics and motion of the specimen in the numerical simulation, the magnetic field physics from the AC/DC module [31] and the deformed mesh physics from the mathematics module were selected. In the numerical analysis, low-carbon steel 1006, low-carbon steel 1002, and copper were selected as the materials for the specimen, ferromagnetic yokes, and Hall-effect sensor, respectively, as shown in Figure 3 and Table 1. Figure 4 shows the BH curves of low-carbon steel 1002 and 1006. Moreover, the surrounding air in the experiment was modeled as an air domain in the numerical model.

**Figure 5:**
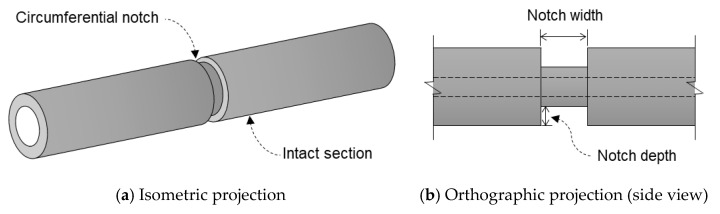
Geometry of the specimen.

In this study, four steel pipe specimens with defects at different depths were modeled, as illustrated in Figure 5. The outer and inner diameters of the specimens were 60 mm and 50 mm, respectively. The defects were modeled as a circumferential notch with the same width of 5 mm and four different depths of 1, 2, 3, and 4 mm.

**Figure 6:**
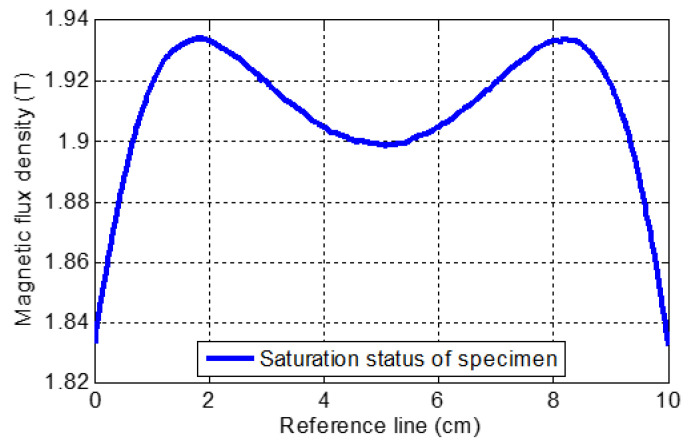
Specimen’s magnetization level for both conventional and proposed numerical simulation.

Based on the MFL inspection theory, an inspection must be conducted when the specimen is either saturated or near saturation [10,30]. As shown in Figure 6, the magnetization level of the specimen was maintained well beyond the saturation point, which is equal to 1.5–1.6 T. Hence, the saturation requirement of the specimen was satisfied for all of the numerical simulations conducted in this study.

### 3.2. Conventional Method

Conventionally, a simple 2D or 3D static simulation is performed to obtain any changes in the MFL from the specimen into the surrounding space in the presence of a flaw. This MFL is obtained by assigning a reference line with a relatively small liftoff right above the defect in the simulation model. It is worth noting that the use of a reference line in air or vacuum may simply disregard the Lorentz force and interaction between the magnetic and electric fields. Consequently, the current induction that appears in a conductive plate (i.e., Hall-effect sensor) is neglected also. In this study, a conventional numerical simulation was conducted by assuming a static simulation over a 2D axisymmetric domain. Defects were modeled as a circumferential notch with the same width equal to 5 mm and four different depths equal to 1, 2, 3, and 4 mm. Moreover, the reference line was defined above the defects with a 5 mm liftoff.

**Figure 7:**
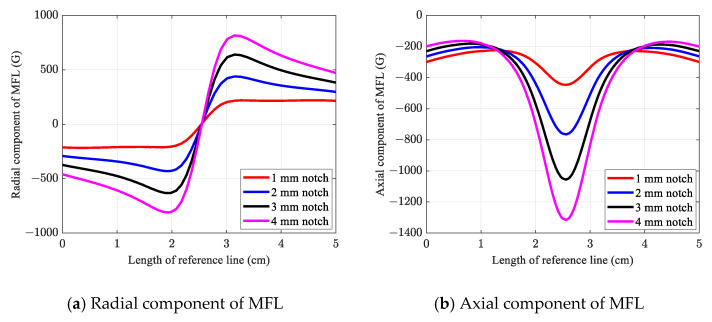
MFL signal obtained from the conventional numerical simulation.

Figure 7a,b illustrate the radial and axial components of the MFL acquired from the reference line in the conventional numerical simulation, respectively. As shown in the figures, all defects could be detected with distinguishable signal patterns and amplitudes in the case of both the radial and axial components of the MFL. In the case of the radial component of the MFL, a sinusoidal response with one peak and one trough corresponding to the notch defect was observed. This nonlinearity is caused by the opposite magnetic polarities induced on the sides of the notch. Thus, MFL has a positive sign on one side of the notch, while it has a negative sign on the other side [18,23]. As expected, shallower notches corresponded to signal amplitudes, whereas deeper defects demonstrated higher signal amplitudes.

Nonetheless, the output signals obtained using the conventional method could only provide information about the MFL, whereas in the experiment, the output signal is directly obtained in either the voltage or current density unit. In the case of circumferential cracks and notches, the radial component of the MFL is the dominant and most effective component of the MFL on the Hall sensor. As a result, from this point, the “radial component of MFL” is referred to as “MFL.”

### 3.3. Proposed Approximate Method

As discussed previously, to obtain the induced current density, rather than the MFL signal, the geometry of the Hall sensor was entered into the simulation model, and time-dependent simulations were performed in the 2D axisymmetric dimension using the proposed method. Compared with the conventional method, these additional steps lead to current induction in the circumferential direction with respect to the inspection direction (i.e., axial direction). The liftoff of the conductive plate, which is representative of the Hall sensor, was assumed to be 5 mm (i.e., the same as conventional numerical simulation). The same specimens used in the conventional method were used again for the proposed method. The difference is that, in this case, the induced current density was obtained when the specimen was passed through the sensing apparatus.

**Figure 8:**
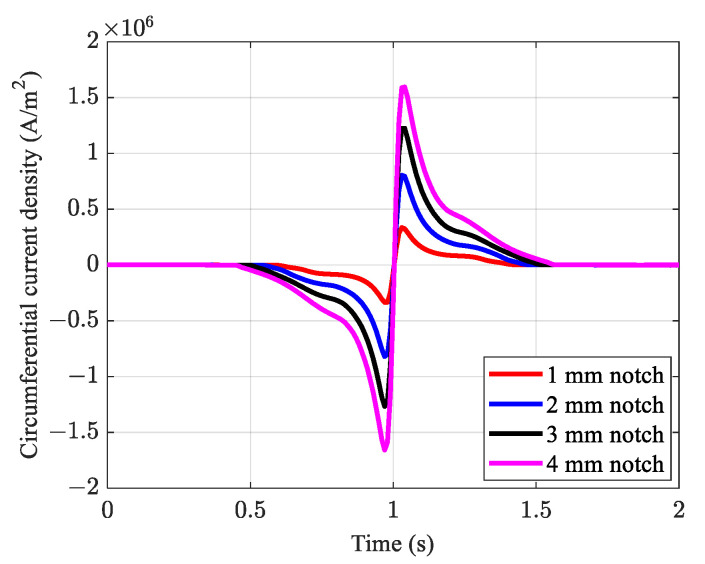
Circumferential current induction obtained using the proposed approximate numerical method.

As shown in Figure 8, all defects were detected with a sinusoidal pattern with only one difference compared to the conventional method. Two peaks and two troughs appeared, rather than having one peak and one trough in the output signal. The main reasons for the extra broad peak and trough with insignificant amplitudes on each side of the defect are the magnetic flux concentration on the edges of the magnets and the slightly raised section of the handles, which provides housing for an array of magnets, and a slightly non-uniform distribution of induced magnetization in the region of interest (Figure 6). Although the specimen was completely saturated in the region of interest, there was still a fluctuation in magnetization, particularly in the vicinity of the circumferential notch, which can ultimately lead to fluctuations in the MFL. This slight magnetic flux fluctuation causes a change in the current density induced in the conductive plate. In addition, for the conventional method, the length of the reference line is limited to the gap between the magnets on each side of the yokes. Moreover, because of the extreme influence of the magnetic field in the vicinity of the permanent magnets, it does not extend to magnets mounted on both sides of the yokes. Unlike the proposed approximate method, by conducting stationary simulations using the conventional method, the velocity term of the specimen is not considered. As observed in the conventional method, in the proposed approximate method, shallower notches corresponded to smaller peak amplitudes, whereas deeper notches had greater peak amplitudes.

### 3.4. Effects of the Liftoff: Comparison between Conventional and Proposed Methods

Generally, to acquire a more intense output signal, the liftoff is chosen to be as small as possible. Evidently, by providing a smaller gap between the surface of the specimen and the sensor tip, a more intense MFL will reach the sensor; therefore, a higher signal amplitude will be obtained. However, the excessive attraction force of permanent magnets does not enable quite small liftoff values. In addition, misdetection may arise when this value is set to be quite large. Under in situ conditions, the liftoff value may not be as consistent as the ideal numerical analysis model. To reflect the significance of liftoff variation effects on the output signal of the Hall sensor, a variety of sensor liftoff values were considered and simulated using both conventional and proposed numerical methods. Thus, five different liftoff values were assumed and modeled: 5, 10, 15, 20, and 25 mm. The same defective specimens used in the previous sections were also used.

**Figure 9:**
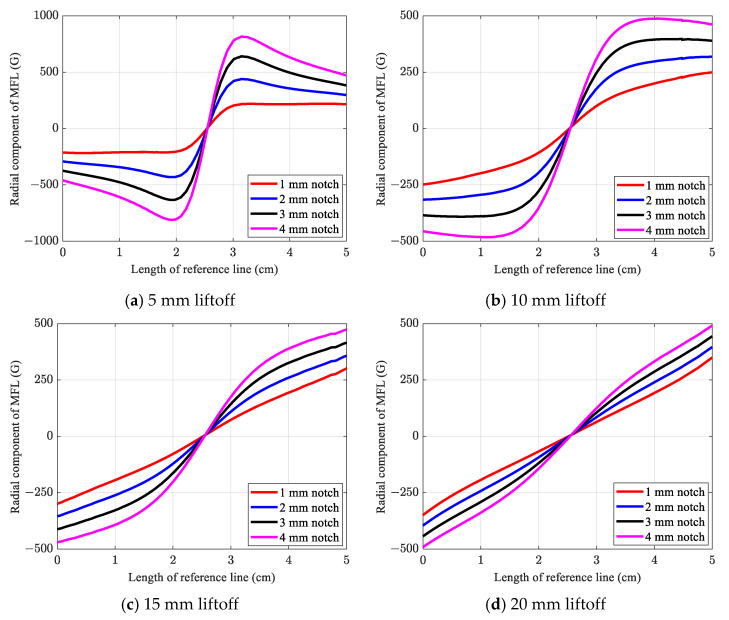

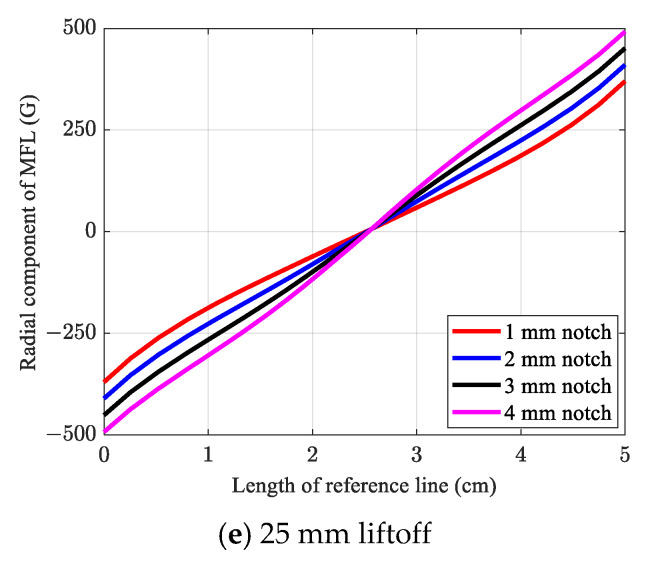
Effects of varying liftoff values on obtained MFL signal using the conventional method.

Figure 9 shows the MFL signals obtained using the conventional numerical method by considering different liftoff values. From the results, when the liftoff is equal to 5 mm, all defects were identified with a distinguishable sinusoidal-shape signal. Furthermore, shallower notches surrendered smaller signal amplitude, while deeper notches demonstrated more intense MFL signals. Meanwhile, by increasing the liftoff value to 10 mm, all defects were detected, except for the notch with a 1 mm depth, and the amplitude of the signals decreased compared to the signals corresponding to the 5 mm liftoff. In the case of a 1 mm notch, which is the shallowest notch, no sinusoidal pattern existed, and it therefore remained almost undetected. From the results, by increasing the liftoff value, it becomes difficult to identify a defect based on the sinusoidal pattern. Clearly, for a liftoff value above 15 mm, it became almost impossible to distinguish the defect patterns of the notches. For instance, with the 20 mm liftoff, except for the notch with a 4 mm depth, all defects had only a linear signal pattern without possessing a sinusoidal pattern.

Furthermore, by increasing the liftoff to 25 mm, all notches remained undetected owing to the absence of a distinguishable defect pattern in the corresponding signals. Thus, the conventional method in this study could only identify all defects, while the liftoff was less than or equal to 10 mm. However, as explained in the next section, the Hall sensor is experimentally proven as capable of detecting defects with a liftoff value greater than 10 mm.

**Figure 10:**
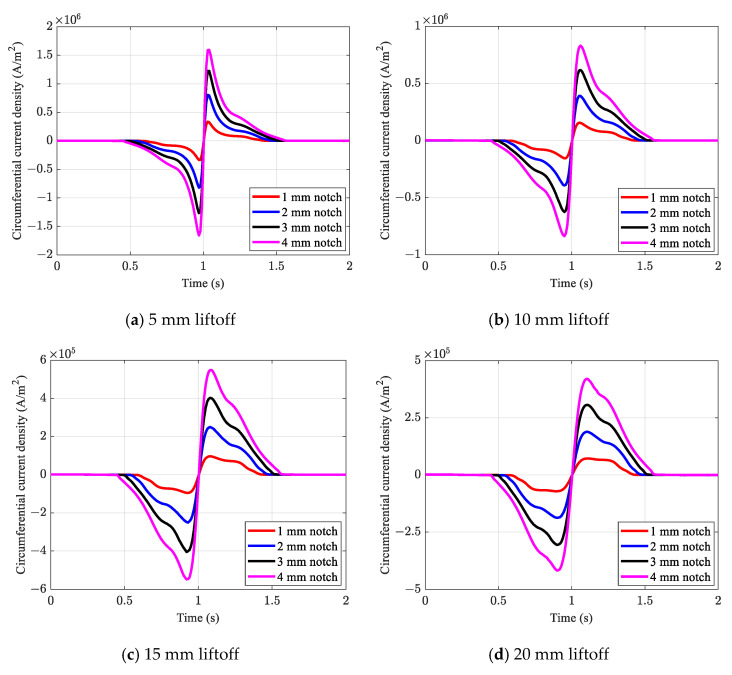

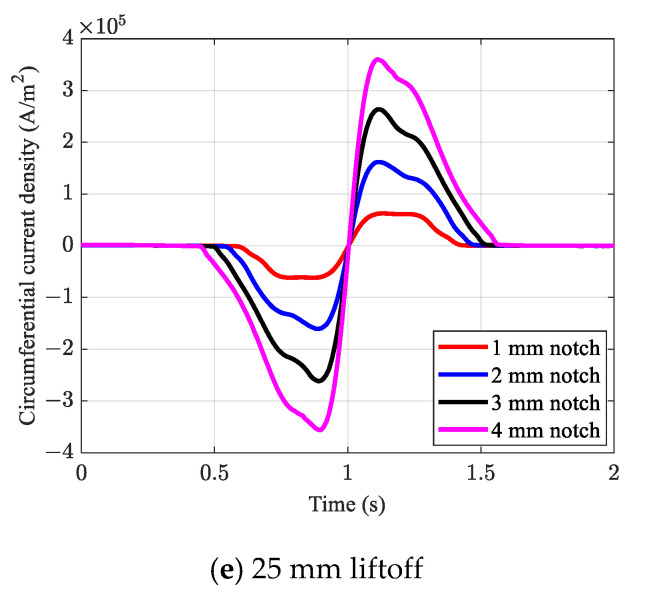
Effects of varying liftoff on circumferential current induction obtained from the proposed method.

The results of the circumferential current induction in the conductive plate based on the proposed approximate numerical method are presented in Figure 10. Unlike the conventional numerical method, the proposed method was capable of detecting all notches, even when considering large liftoff values that are well beyond the threshold of the conventional numerical method. As shown in Figure 10, in the case of a 10 mm liftoff, all defects were identified with sharp and distinguishable signal patterns. Although the signal amplitude decreased in the case of a 25 mm liftoff, even the shallowest defect presented a distinctive defect pattern.

## 4. Experimental Validation

### 4.1. Methods Description of the Test Setup

**Figure 11:**
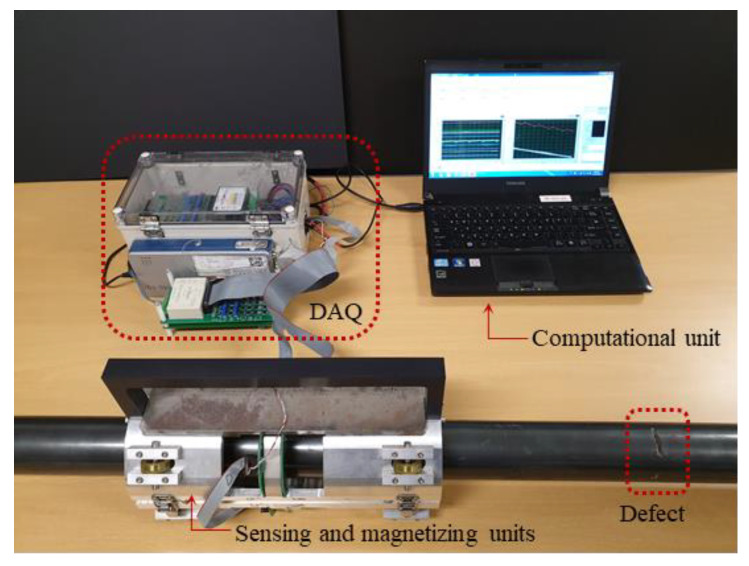
Electromagnetic sensor, defected specimen, and data acquisition (DAQ) system.

**Figure 12:**
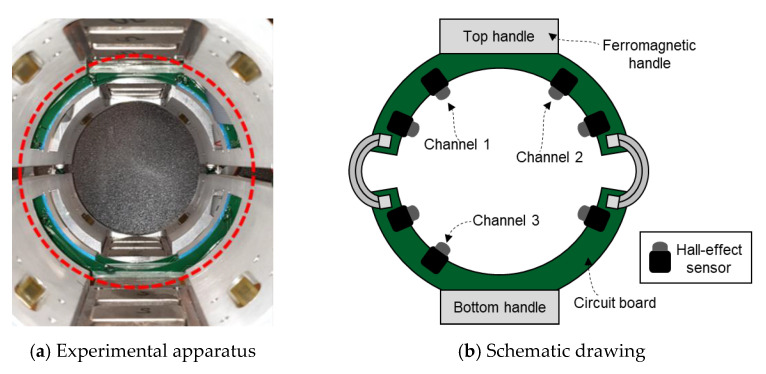
Array of the Hall-effect sensors.

In this study, a customized electromagnetic system was used to perform experimental validation. The inspection system consists of sensing and magnetizing units as well as a data acquisition system, as shown in Figure 11. Twelve neodymium magnets were symmetrically embedded on two ferromagnetic handles, six each on the top and bottom handles in the magnetic apparatus (i.e., in each yoke, six magnets were placed symmetrically). On the circuit board placed in the middle of the magnetic apparatus, seven HW-300A Hall-effect sensors were circumferentially placed, as illustrated in Figure 12. The entire setup was held together using an aluminum frame. To acquire the output signal of the Hall-effect sensor, this study used an NI cDAQ-9181 Ethernet compact data acquisition chassis with an NI 9205 analog input terminal module that has a sampling rate of 500 Hz.

**Figure 13:**
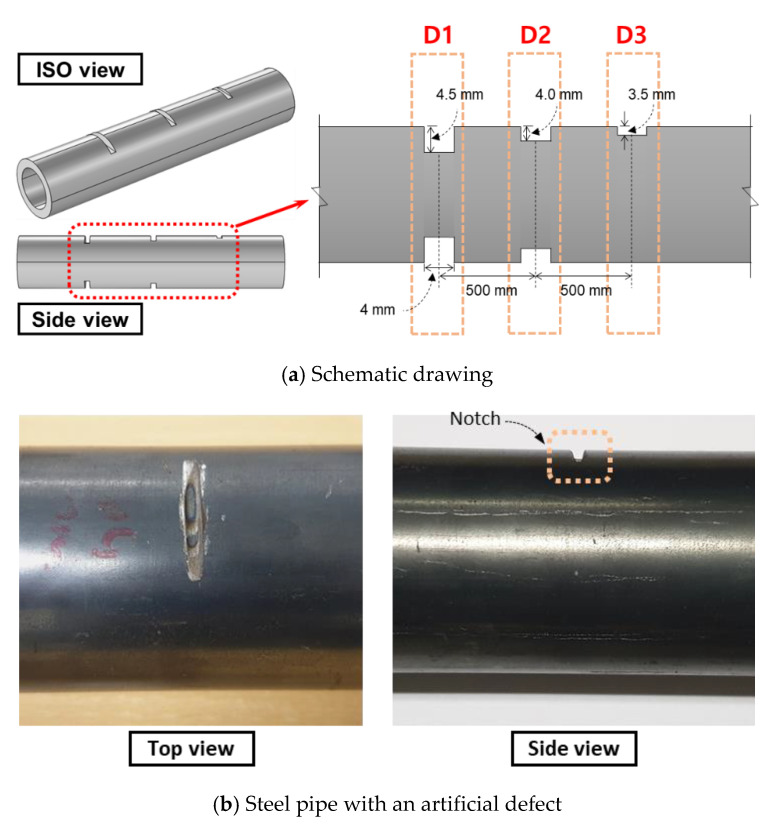
Defected specimen.

For testing, a hollow steel pipe was prepared with outer and inner diameters of 60 and 50 mm, respectively. Five artificial notches of different depths were fabricated on the pipe. As shown in Figure 13 and Table 2, these five notches were labeled as three sets of defects: D1, D2, and D3. Here, D1 has two notches with a depth of 4.5 mm, D2 has two notches with a depth of 4 mm, and D3 has one notch with a depth of 3.5 mm located on the top of the specimen. Here, the width of each notch was 4 mm, and they were equally spaced 500 mm apart.

To address the liftoff variation issue and its effects on the output signal, experimental tests were designed by considering various liftoff configurations. As shown in Table 3, four different liftoff configurations were designed. Hence, for the top sensors located in the vicinity of the top handle (i.e., channels 1 and 2), the offsets were equal to 30, 25, 20, and 15 mm in test cases C1, C2, C3, and C4, respectively, and the exact liftoffs were equal to 39, 35, 32, and 29 mm in the C1, C2, C3, and C4 test configurations, respectively. In the case of the bottom sensor located near the bottom handle (i.e., channel 3), the offsets were equal to 20, 25, 30, and 35 mm in test cases C1, C2, C3, and C4, respectively, and the exact liftoffs were equal to 32, 35, 39, and 43 mm in the C1, C2, C3, and C4 test configurations, respectively. To observe the change in potential differences for each test case, the pipe specimen placed inside the magnetic apparatus was manually extracted while maintaining the pulling speed constant.

### 4.2. Experimental Results

**Figure 14:**
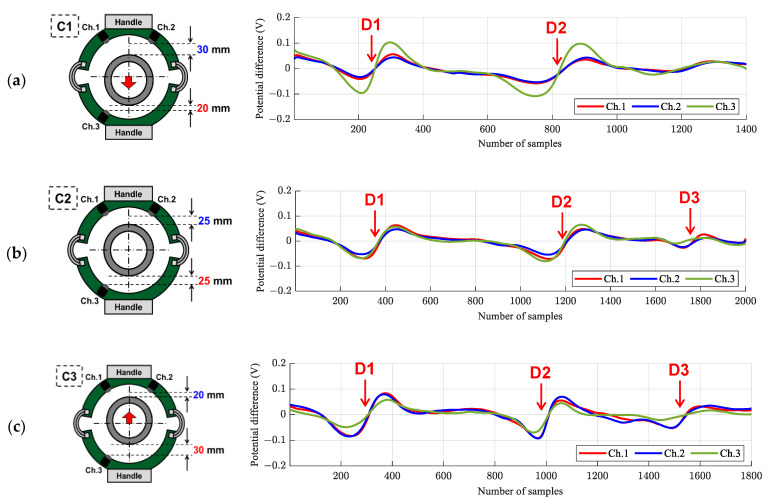

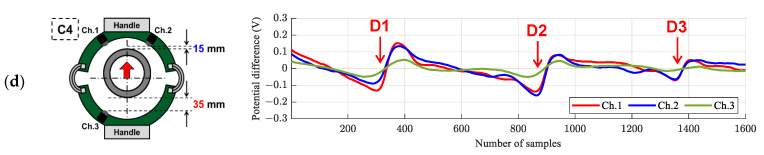
Effects of the liftoff variation on the Hall voltage obtained from the experimental apparatus: (**a**) C1 configuration with 30 and 20 mm offsets from top and bottom sensors, respectively; (**b**) C2 configuration with 25 mm offset from top and bottom sensors; (**c**) C3 configuration with 20 and 30 mm offsets from top and bottom sensors, respectively, and (**d**) C4 configuration with 15 and 35 mm offsets from top and bottom sensors, respectively.

Figure 14 and Table 4 show the representative experimental results of the potential differences obtained from the defective pipe specimen. In the figure, each row represents the results for a different liftoff configuration (i.e., C1, C2, C3, and C4). The schematics given on the left side of the figure represent the specimen’s locations and corresponding liftoffs, and the graphs on the right represent the potential differences. The red, blue, and green solid lines indicate the potential differences obtained by channels 1, 2, and 3, respectively. Here, the *x*-axis represents the number of samples collected at a sampling rate of 500 Hz.

As discussed previously, defects on metal specimens were detected with a sinusoidal pattern in the proposed numerical approach. Similarly, in the experiments with the magnetic apparatus, the defects were also detected with a sinusoidal pattern with potential differences.

When the specimen was located at the center of the magnetic apparatus (C2) shown in Figure 14b, the distance between the specimen and each Hall sensor channel (i.e., 1, 2, and 3) was 35 mm. Hence, the liftoff value was 35 mm for all channels. In this case, D1 and D2 were detected with a distinctive sinusoidal pattern by channels 1, 2, and 3, as shown in Figure 14b. The maximum potential differences were in the range of approximately 0.12 V for D1 and D2, but the amplitudes were slightly different compared to each other. Unlike the defects located in both the upper and lower parts of the sample (D1 and D2), the D3 defect consisted of only one notch located in the upper part, which was detected by channels 1 and 2, and its amplitude (0.06 V) was distinctively smaller than D1 and D2. Meanwhile, no significant differences were observed between channels 1 and 2.

The changes in the potential difference were observed when the specimen was not in the center of the magnetic apparatus. First, the specimen was moved downward by 5 mm from the center (C1) to change the liftoffs, as shown in Figure 14a. In this configuration, the liftoffs of channels 1, 2, and 3 were 39, 39, and 32 mm, respectively. In this case, D1 and D2 were detected in both the top- (Ch. 1 and 2) and bottom (Ch. 3)-located Hall sensors; however, the D3 defect was not detected by either the top or bottom Hall sensors. Here, owing to the smaller gap between the specimen and the bottom-located sensor (Ch.3) than the top-located sensors (Ch. 1 and 2), the signal obtained from channel 3 had a larger amplitude (0.2 V), compared to that of the top-located sensors (0.1 V).

In the same manner mentioned previously, the specimen was moved upward by 5 mm from the center (C3), as shown in Figure 14c. In this configuration, the liftoff value for channels 1, 2, and 3 were 32, 32, and 39 mm, respectively. D1 and D2 were detected in all sensors, but their corresponding signal amplitudes were different. Hence, the top-located sensors had a higher signal amplitude (0.16 V) owing to their smaller liftoff values compared to the bottom-located sensor (0.10 V). In the case of D3, owing to the large distance between channel 3 and the defect, the defect was detected only in the top-located sensors.

In test case 4 (C4), the specimen was moved upward 10 mm from the center, as shown in Figure 14d. Thus, the liftoff values of channels 1, 2, and 3 were 29, 29, and 43 mm, respectively. Similar to C3, D1 and D2 were detected in all sensors, but their corresponding signal amplitudes were different. Hence, owing to the noticeable difference in liftoff between the top and bottom sensors, the difference in amplitude between the top (0.25 V) and bottom (0.09 V) sensors became significantly larger than that of the C3 test setup.

Based on the results acquired by the test setups for C1, C2, C3, and C4, it was found that D3 could be identified with liftoff values less than or equal to 35 mm. Moreover, D1 and D2 were detected for all varieties of liftoffs; however, by increasing the liftoffs, the signal amplitudes started to decrease noticeably. Unlike the results obtained using the conventional numerical method, which was limited to a 10 mm liftoff, in the experimental test, even with large liftoff values (e.g., 32 and 35 mm), all defects were successfully identified by Hall-effect sensors.

Although the potential differences obtained by channels 1 and 2 should be theoretically equal, it was observed that the experimental results were not the same. This is because the potential differences were obtained while pulling the specimen out from the magnetic apparatus manually (i.e., using bare hands) in this study; that is, the slight potential difference between channels 1 and 2 resulted from the inaccurate position of the specimen in the magnetic apparatus. In addition, owing to the slightly unequal velocity of the specimen during inspection, defect patterns were observed within different time periods for each test configuration (C1, C2, C3, and C4). Despite that defect patterns were observed within different time periods, owing to the Hall-effect phenomenon, the inconsistent velocity of the specimen had no significant influence on the signal amplitudes.

## 5. Conclusions

This study aimed to address the liftoff variation issue and its effects on the output signal of the Hall-effect sensor through both numerical simulation and experimental tests. To this end, variations in the liftoff and the corresponding effects on notch defects having different depths were assessed. The simulation results indicate that the conventional numerical simulation method was incapable of detecting defects in two cases: shallow notches with small liftoffs and both shallow and deep notches with liftoffs greater than 10 mm. Conversely, the proposed approximate numerical method can identify all defects with distinctive sinusoidal patterns well beyond the 10 mm threshold liftoff of the conventional method. Moreover, unlike the conventional method, no misdetection occurred even in the case of a 25 mm liftoff and when the shallowest notch existed.

This indicates that the conventional numerical simulation method can only detect defects within a limited range of liftoff values. Conversely, the proposed semi-precise analytical method can identify defects with no difficulties beyond this threshold. Finally, the effects of liftoff variation were investigated experimentally. The experimental test results indicate that a distinctive defect pattern existed in the signals with liftoff values higher than the threshold of the conventional numerical method. In addition, the detectability of the proposed approximate method beyond the threshold of the conventional method was confirmed.

According to four different test configurations, all defects were identified when the liftoff value was less than or equal to 35 mm. In addition, deep defects were detectible even with liftoff values greater than 35 mm. Based on the results obtained by both numerical and experimental tests, it can be concluded that the conventional numerical method cannot provide a reasonable estimation of the liftoff requirement. Conversely, the proposed approximate method provides a better estimation. However, it should be noted that only one type of defect (i.e., notch) was considered in this study. Therefore, the findings here cannot be generalized for other applications. Furthermore, to improve the reliability and precision of the proposed numerical method, there is the need for further studies to propose a precise numerical approach based on Hall voltage induction.

## Figures and Tables

**Table 1:** Material properties.

Object	Material	Electrical Conductivity (MS/m)	Relative Permeability
Air	Air	0	1
Hall-effect sensor	Copper	59.98	1
Ferromagnetic yoke	Low-carbon steel 1002	8.41	(BH curve was used)
Specimen	Low-carbon steel 1006	8.41	(BH curve was used)

**Table 2:** Details of the defect cases.

Defect Case	Width	Depth		Location	
		Maximum Notch Depth	Increment	At Specimen	Nearest Sensor
D1	4.0 mm	4.5 mm	−0.5 mm (↓)	Top and bottom	Ch. 1, 2, 3
D2	4.0 mm	4.0 mm		Top and bottom	Ch. 1, 2, 3
D3	4.0 mm	3.5 mm		Top	Ch. 1, 2

**Table 3:** Liftoff configurations in the vertical axis direction (*z*-axis).

Test Case	Location of Specimen from the Center of Experimental Apparatus		Liftoff Configuration		
			Reference Point	Offset	Liftoff
C1	−5 mm	Increment: +5 mm (↓)	Ch. 1 (Ch. 3)	30 mm (20 mm)	39 mm (32 mm)
C2	0 mm		Ch. 1 (Ch. 3)	25 mm (25 mm)	35 mm (35 mm)
C3	+5 mm		Ch. 1 (Ch. 3)	20 mm (30 mm)	32 mm (39 mm)
C4	+10 mm		Ch. 1 (Ch. 3)	15 mm (35 mm)	29 mm (43 mm)

**Table 4:** Experimental results.

Sensor Number	Test Case	Liftoff Configuration		Defect Detection Result (Maximum Potential Difference)		
		Offset Distance	Liftoff Distance	D1	D2	D3
Ch. 1 & Ch. 2	C1	30 mm	39 mm	Detected (0.10 V)	Detected (0.10 V)	Missed
	C2	25 mm	35 mm	Detected (0.12 V)	Detected (0.11 V)	Detected (0.06 V)
	C3	20 mm	32 mm	Detected (0.16 V)	Detected (0.15 V)	Detected (0.08 V)
	C4	15 mm	29 mm	Detected (0.25 V)	Detected (0.23 V)	Detected (0.14 V)
Ch. 3	C1	20 mm	32 mm	Detected (0.20 V)	Detected (0.20 V)	Missed
	C2	25 mm	35 mm	Detected (0.11 V)	Detected (0.13 V)	Missed
	C3	30 mm	39 mm	Detected (0.10 V)	Detected (0.11 V)	Missed
	C4	35 mm	43 mm	Detected (0.09 V)	Detected (0.08 V)	Missed

## Data Availability

Not applicable.
